# Waste Surgical Masks as Precursors of Activated Carbon: A Circular Economy Approach to Mitigate the Impact of Microplastics and Emerging Dye Contaminants

**DOI:** 10.3390/ma18174115

**Published:** 2025-09-02

**Authors:** María del Mar García-Galán, Carlos A. Fernández-Blanco, Eduardo M. Cuerda-Correa, Juan M. Garrido-Zoido, María F. Alexandre-Franco

**Affiliations:** 1 Departamento de Dirección de Empresas y Sociología, Universidad de Extremadura, Avenida de Elvas s/n, 06006 Badajoz, Spain; margalan@unex.es; 2 Departamento de Química Orgánica e Inorgánica, Facultad de Ciencias, Universidad de Extremadura, Avenida de Elvas s/n, 06006 Badajoz, Spain; cfernandaf@alumnos.unex.es (C.A.F.-B.); emcc@unex.es (E.M.C.-C.)

**Keywords:** surgical mask, activated carbon, methylene blue, methyl orange, orange G, circular economy, carbonaceous adsorbent materials, polluting dyes

## Abstract

The COVID-19 pandemic has caused a surge in the use of disposable surgical masks, primarily composed of polypropylene (>86% carbon), whose improper disposal contributes to persistent microplastic pollution. In alignment with circular economy principles, this study explores the valorization of surgical masks into carbonaceous adsorbent materials (ACMs) for dye removal from water. The masks were chemically treated with concentrated H_2_SO_4_ at 85 °C for 2 h and subsequently activated with air (400 °C), CO_2_, or steam (800 °C, 1 h). The resulting ACMs were characterized by SEM, FT-IR, nitrogen adsorption at −196 °C, and pH of the aqueous carbon suspension (pH_Sus_, 1.96–9.25). CO_2_ and steam activation yielded the highest surface areas (525 and 632 m^2^·g^−1^, respectively). FT-IR confirmed the introduction of sulfonic groups, enhancing dye interactions. Adsorption tests using methylene blue (MB), methyl orange (MO), and orange G (OG) in ultrapure and river water showed removal efficiencies up to 100% for MB with ACM-WV and ~94% with ACM. All dyes followed pseudo-second-order kinetics. These findings demonstrate that surgical mask waste can be effectively transformed into high-value adsorbents for water treatment applications.

## 1. Introduction

The circular economy is an economic model aimed at minimizing waste and maximizing the use of resources [1,2]. Unlike the traditional linear economy (take–make–dispose), the circular economy focuses on closing the loop through strategies such as reduce (minimizing resource use and waste generation), reuse (extending the life of products and components), recycle (recovering materials to make new products), repair and refurbish (restoring used items for further use), and design for longevity (creating products that last longer and are easier to maintain, upgrade, or recycle). This model benefits the environment by reducing pollution and resource depletion, and it supports economic resilience by creating new business opportunities and jobs in areas like recycling, remanufacturing, and sustainable design [1,2].

Governments, companies, and consumers all play key roles in shifting toward a circular economy by adopting sustainable practices, designing smarter products, and supporting circular business models.

Among the many sectors impacted by this paradigm shift, the textile industry stands out for its significant environmental footprint and active engagement in circular practices. The global textile sector is strongly focused on applying the concept of the circular economy within its supply chains. In fact, major companies such as Inditex, H&M, and Gap have already initiated this process [3].

The production of textile fibers is one of the largest global industries, with an increasing production of over 100 million tonnes, which is expected to rise to 160 million tonnes in 2030 if current trends continue [4]. It has been noted that the market value exceeds USD 1 trillion [5]. Textile waste is a relevant problem that can be classified into three main types: pre-consumer, post-industrial, and post-consumer [6]. Some of these issues include large-scale water consumption and land use, their pollution, and the amount of CO_2_ released into the atmosphere. Of interest for the present work is the post-consumer waste that represents textiles after their use by end consumers and, particularly, is often contaminated by dyes [7], which are considered emerging contaminants in water. Dyes are organic molecules with two components: the chromophore, consisting of conjugated double bonds, with resonance of electrons and enabling the absorption of light; and the auxochromes, electron-accepting or electron-donating groups that increase the affinity of the dye molecule for the fiber and modify the light absorption [8,9,10].

While post-consumer textile waste and dye contamination pose one set of environmental challenges, another emerging issue exacerbated by the pandemic is the accumulation of disposable surgical masks. Although facial masks were first used in the 14th century, becoming a protection mechanism against any bacterial or viral threat, and even protection against atmospheric agents, with the spread of COVID-19, they have really taken off and have become part of our daily lives. In fact, their demand is expected to continue to grow globally in the coming years. The environmental pollution caused by the use of facial/surgical masks is due to their persistence and accumulation. The surgical mask is mainly composed of polypropylene, which slowly degrades into smaller particles, giving rise to a new source of microplastics that are considered a new type of environmental pollutant. The extensive use of surgical masks constitutes a serious pollution problem, and it has generated a significant amount of plastic waste in the environment.

When these particles come into intimate contact with the soil, they can cause changes in soil bulk density, water holding capacity, among others [11]. In addition, these microplastic particles together with other contaminants are transferred from the topsoil to a deeper layer with the help of tillage and leaching activities into the groundwater. This affects the development of plants since small plastics can enter the plant through absorption and thus enter the fauna. The accumulation of disposable masks can also pose a serious risk, as aquatic organisms may ingest them and mistake them for plankton. Numerous studies have detected the presence of microplastics in the digestive tract of many marine species, such as mollusks, cetaceans, etc. [12].

In addition, contaminants carried by microplastics can be transported through the food chain to humans [13]. Among the chemicals present in them are heavy metals (chromium, copper, nickel, lead, and zinc) [12] that can form complexes with reactive dyes. The complexes formed have been found in saliva and sweat droplets, which can be considered a transport mechanism into the human body. In addition, these complexes associated with microplastics resulting from mask release can be transferred and enter the respiratory tract by inhalation or skin contact.

Recent studies have indicated that plastics accumulate in areas of the human body, such as the lungs, which are very difficult to degrade and, thus, eliminate over the course of a human lifetime [14]. Ultimately, both the disposable respirators themselves and the microplastic particles resulting from their release pose a serious threat to the ecosystem as they are not susceptible to assimilation back into nature. It is therefore essential to explore effective treatments to minimize microplastic pollution at source [15].

Addressing this challenge requires not only waste management strategies but also an in-depth understanding of the materials involved and their potential for transformation. Efforts are currently directed towards the study of the relationships between the structure and properties of materials, as well as the development of production processes that improve their quality, competitiveness, and compatibility with environmental conservation. Polymers are among the most versatile materials and compare favorably with other classical materials such as steel, aluminum, and ceramics, which offer excellent properties together with significant advantages in weight, processing, and price. Among the polymers, polyolefins constitute an important family of materials, in which polyethylene (PE) and polypropylene (PP) are widely used in industry due to their characteristics: low cost, good mechanical, electrical and chemical resistance, good optical properties, ease of processing and recycling, reaching a world production share of more than 65% of total plastics, which is expected to continue growing over the next few years [16].

Scientific work on the contamination of facial masks is focused on the valorization of this material. According to the 5R rule, the most important steps are reuse and recycle. There are methods such as burning the masks and harnessing the energy that can be generated. More recently, the reuse of facemasks has shifted toward nanotechnology, which primarily involves the use of antimicrobial/antiviral nanoparticles, nanofibers, and nanoparticle coatings to achieve super-hydrophobicity [17].

The recycling of masks is a viable measure from both a technical and environmental point of view, as it yields materials with significant added value and helps reduce the generation of persistent environmental waste. In fact, as mentioned above, plastic waste is not susceptible to being assimilated back into nature. Furthermore, it has been demonstrated that disposable face masks can filter chemical pollutants and microfibers into the environment under experimental conditions.

Activated carbon (AC) exhibits excellent textural and surface chemical properties; that is, a high degree of surface development and porosity, as well as functional groups and surface structures. Owing to these features, AC has numerous applications. Its properties depend on the raw material used and on the preparation method and conditions. Common industrial precursors include wood, shells, bones, peat, lignite, and coal [18]. However, a current trend in AC production is the use of abundant waste or residual materials that presently lack economically viable applications. Currently, many reported biomass raw materials have been extensively used to produce activated carbon [19]. For example, wood waste demonstrates high adsorption capacity toward organic contaminants [20,21], while agro-industrial residues have been successfully valorized into activated carbons for dye removal in industrial wastewater [22,23]. Moreover, agricultural and forest wastes are being upcycled into biochar for environmental applications, including industrial wastewater treatment and air-pollution control [19,24,25,26].

Utilizing such materials not only promotes their valorization but also plays a key role in mitigating a persistent environmental pollution problem. In this context, identifying new precursors that are both carbon-rich and abundant in waste streams is essential. The large-scale generation of discarded face masks—estimated at 5.7 to 8.5 trillion units annually, depending on the daily usage rate—offers a promising opportunity for their conversion into high-value carbonaceous adsorbents.

Given this environmental concern and the composition of surgical masks—primarily made of polypropylene (approximately 80%), a polymer with a high carbon content (over 86%), a nonwoven textile (TNT) rich in carbon—they are considered suitable precursors for the production of activated carbon. Thus, for example, the potential of porous carbon materials derived from face masks has been investigated for use in lithium-sulfur batteries [27].

In addition to adsorption, other approaches for dye removal from surface waters have been explored in recent years, including advanced oxidation processes [28,29], membrane filtration [30,31], and coagulation–flocculation methods [32,33]. While these techniques can be effective, they are often associated with high operational costs, secondary pollution, or limited efficiency in complex water matrices. Adsorption onto activated carbons remains one of the most widely applied and versatile methods, combining high efficiency, relatively low cost, and straightforward operation, which reinforces the relevance of developing sustainable precursors such as discarded surgical masks.

Building on this premise, the present research explores how the transformation of surgical masks into activated carbon could contribute to both waste valorization and water purification. Interestingly, as early as 1854, activated carbon was already being used as an adsorbent in personal protective equipment (PPE), specifically in protective masks for miners. In a circular turn, this study proposes using another type of PPE—surgical masks—as a starting material for producing activated carbon, aligning with the principles of a circular economy.

## 2. Materials and Methods

### 2.1. Chemicals and Materials

The starting material used for the preparation of carbonized and activated samples was surgical masks (SM) manufactured by PI MEDICAL LABS S.L., located in Don Benito, Badajoz, in the Extremadura region of Spain. The nasal wires and corresponding elastic bands were removed from the surgical masks. Subsequently, they were cut into pieces of approximately 1 × 1 cm. In this proof-of-concept study, only new, unused surgical masks were employed to eliminate any potential biological hazard during laboratory handling. We acknowledge, however, that real-world valorization would require the safe processing of used masks. In such cases, standardized sterilization methods (e.g., autoclaving, dry heat, or chemical disinfection) should be applied before carbonization to ensure biosafety for operators and suitability of the material for further activation.

Methylene blue, methyl orange, and orange G from the commercial supplier Sigma-Aldrich (Merck Life Science S.L.U., Madrid, Spain) were used as adsorbates. N_2_ (gas, quality N-48), ALPHAGAZ synthetic air, and CO_2_ were supplied by the company Air Liquide (Madrid, Spain). Sulfuric acid was purchased from the commercial supplier Labkem (Barcelona, Spain) with a purity of 95–97%, and the potassium bromide for infrared spectroscopy was from the Merck brand (Merck Life Science S.L.U., Madrid, Spain). Ultrapure Milli-Q water and Guadiana River (Badajoz, Spain) water were used as the aqueous matrices.

### 2.2. Preparation of Carbonaceous Adsorbent Materials

The chemical treatment was carried out by immersing 100 g of SM in a beaker containing 300 mL of concentrated sulfuric acid, followed by heating at 85 °C for 2 h, until a viscous residue remained, with a supernatant acid phase. The use of H_2_SO_4_ in this initial step was justified by its ability to carbonize polypropylene fibers, promote partial sulfonation, and introduce oxygen-containing functional groups on the surface. In addition, the treatment removes impurities and facilitates the formation of a more reactive structure, thereby enhancing the efficiency of the subsequent physical activation processes. To promote the action of sulfuric acid on the starting material, magnetic stirring was employed to ensure thorough mixing. Once cooled, the solid residue was filtered and washed with ultrapure water until the liquid reached a circumneutral pH. The sample was then dried in an oven at 60 °C for 12 h, followed by drying at 120 °C for 24 h. After cooling in a desiccator, the sample was weighed to calculate the overall yield of the process. Finally, the sample was stored in hermetically sealed containers. The notation used for this sample was ACM.

The preparation of the activated carbon from the ACM samples was carried out by the physical activation method with atmospheres of air, carbon dioxide, and steam. A CARBOLITE horizontal furnace (Carbolite Gero Ltd., Hope Valley, UK) with a 60 mm diameter and a 65 cm long ceramic tubular reactor was used for activation with air and CO_2_. An IBERLABO furnace (Iberlabo S.L., Madrid, Spain) was used for steam activation. The air-activated carbon sample (ACM-A) was obtained by maintaining a temperature of 400 °C for 2 h. The carbon samples activated with CO_2_ (ACM-CO_2_) and with water vapor (ACM-WV) were maintained at 800 °C for 1 h. Air activation was carried out at 400 °C for 2 h to promote controlled surface oxidation and the formation of oxygen-containing functionalities, while avoiding fast combustion and excessive mass loss. Activation with CO_2_ and steam at 800 °C for 1 h was chosen because at this temperature the C-to-CO_2_ (Boudouard) and C-to-H_2_O reactions display sufficiently high rates to develop microporosity (CO_2_) and expand pores toward the mesopore range (steam), respectively, within a practical duration and without over-gasifying the carbon matrix [34,35].

### 2.3. Characterization of Starting Material and ACMs

The starting material and the various samples prepared, as described previously, were characterized from a physicochemical perspective, providing valuable information about their composition, texture, and the chemical nature of the functional groups present on their surface.

#### 2.3.1. Chemical Characterization

Elemental analysis was carried out at the Research Support Center of the University of Málaga (SCAI-UMA). A TruSpec micro CHNSO analyzer (LECO Instrumentos S.L., Tres Cantos, Spain) was used, based on the total combustion technique applied to 2–10 mg of sample, and equipped with independent detectors for each element: a non-dispersive solid-state IR detector for C, H, and S, and a differential thermal conductivity detector for N. Oxygen analysis was performed using a separate module of the instrument.

Ash content was determined using a conventional muffle furnace. Approximately 0.1 g of sample was heated in air at 650 °C for 12 h, which are suitable conditions to ensure complete incineration of the sample and conversion of all inorganic matter into ash. Each sample was analyzed in triplicate, and the results were averaged.

The determination of total carbon was carried out using 0.1 g of sample carbon dispersed in 20 mL of distilled water inside a sealed screw-cap test tube. The suspension was maintained at 25 °C under mild agitation (50 rpm) for 7 days in a thermostatic orbital bath, Selecta Unitronic C (J.P. Selecta, Abrera, Spain). After this period, the mixture was filtered, and the supernatant was collected. Aliquots of the filtrate (2 mL dissolved in 10 mL of water and 6 mL dissolved in 6 mL of water) were divided into two sets: one left unacidified to measure total carbon (inorganic + organic), and the other acidified with HClO_4_ and degassed for 3 min to remove inorganic carbon as CO_2_(g), thereby allowing the quantification of organic carbon only. Samples were analyzed in an infrared-based carbon analyzer equipped with a catalytic combustion system. The CO_2_(g) generated was measured to determine the carbon content. The difference between total and organic carbon values allowed the estimation of inorganic carbon.

#### 2.3.2. Morphological Characterization

Scanning Electron Microscopy (SEM) is primarily used for surface observation of materials, providing information on the texture, size, and morphology of the analyzed sample. Sample analysis was carried out in the SCAI-UMA using a Field Emission Scanning Electron Microscope with a Focused Ion Beam (FESEM-FIB) Helios Nanolab 650 model (Thermo Fisher Scientific, Madrid, Spain).

#### 2.3.3. Thermogravimetric Analysis

Thermogravimetric analysis of the starting material was performed at SCAI-UMA using a METTLER TOLEDO thermal analyzer, model TGA/DSC 1 (METTLER TOLEDO International Inc., Cornellà de Llobregat, Spain). This instrument allows simultaneous measurement of Thermogravimetric (TG) and Differential Scanning Calorimetry (DSC) data for sample amounts as low as 10 µg (5 mg were used in our case), enabling the assessment of changes in the physical properties of chemical compounds and materials as a function of temperature or time. The instrument is operated, and data are evaluated using the METTLER TOLEDO STARe system software, version 15.01.

#### 2.3.4. Textural Characterization

N_2_ adsorption isotherms at −196 °C were measured using a semi-automated Autosorb-1 apparatus (Quantachrome Instruments, Boynton Beach, FL, USA). Prior to analysis, the sample was dried in an oven at 120 °C for 12 h and then cooled to room temperature in a desiccator with CaCl_2_. The N_2_ adsorption analysis measures the volume of gas adsorbed as a function of the relative pressure (*p*/*p*^0^, where p is the equilibrium pressure and *p*^0^ is the saturation pressure of the gas at the adsorption temperature). The instrument automatically introduces doses of gas and adjusts the amount injected until the programmed pressure is reached. The adsorbed volume is calculated as the difference between the total volume introduced and the residual volume in the system. This process is repeated to record the different points of the isotherm at increasing relative pressures. Micropore volume (V_mi_) is determined through the adsorption isotherms as the volume of N_2_ adsorbed at *p*/*p*^0^ equal to 0.1. Mesopore volume (V_me_) is calculated using the equation:

V_me_ = V_0.95_ − V_mi_, (1)

where V_0.95_ is the volume of nitrogen adsorbed at a relative pressure of 0.95 [36]. Both V_mi_ and V_me_ are expressed as liquid volumes, using a conversion factor of 1.543 × 10^−3^.

The Brunauer–Emmett–Teller (BET) method was applied to determine the specific surface area (S_BET_) [37], whereas the Dubinin–Radushkevich equation was utilized to compute the micropore volume (W_0_) [38].

The average pore width (APW) was calculated from the nitrogen adsorption isotherms using the Barrett–Joyner–Halenda (BJH) model [39].

Mercury porosimetry provides information on the total porosity and pore size distribution of a solid in the mesopore and macropore range, by recording the volume of mercury intruded as a function of the applied pressure. Mercury intrusion measurements were performed using an Autoscan-60 porosimeter (Quantachrome, Boynton Beach, FL, USA) with 0.5 g of sample previously dried in an oven at 120 °C for 12 h. The mercury displacement densities of the samples were determined as the mass-to-volume ratio, with the volume obtained by mercury displacement during the porosimetry analysis.

#### 2.3.5. Chemical–Surface Characterization

The prepared ACMs were analyzed by infrared spectroscopy using a Jasco Analítica FTIR spectrometer, model 6800FV (JASCO Analítica Spain S.L., Madrid, Spain). Measurements were performed in transmission mode with the samples dispersed in KBr pellets. Spectra were acquired with a standard spectral resolution of 4 cm^−1^ over the range of 4000–400 cm^−1^, using 64 scans per sample. Air was used as the background.

For the determination of the pH at the point of the aqueous suspension (pH_Sus_), a 0.1 M aqueous solution of NaNO_3_ was prepared. Each carbon sample was mixed with an amount of NaNO_3_ equivalent to 7 wt% of the sample and placed in screw-cap test tubes. Then, 10 mL of the NaNO_3_ solution was added to each tube, which were tightly sealed to prevent evaporation. The tubes were placed in a thermostatic orbital bath (Selecta Unitronic Orbital C) at 25 °C and 50 rpm for 48 h. After this period, the suspension was filtered and the pH of the supernatant was measured in duplicate using a Mettler Toledo SevenEasy pH-meter equipped with an InLab Pro electrode (Mettler-Toledo S.A.E., L’Hospitalet de Llobregat, Spain), previously calibrated with three buffer solutions. The measured pH corresponds to the point of the aqueous suspension (pH_Sus_). The pH of the aqueous suspension indicates the surface acidity/basicity of the carbons, which can still influence dye adsorption, although this parameter does not strictly correspond to the point of zero charge (pHpzc).

It should be noted that advanced surface-sensitive techniques such as XPS were not employed in this study. While XPS could provide additional information about the surface chemistry of activated carbons, its application is challenging due to the intrinsic roughness and heterogeneity of highly porous materials and the complexity of peak deconvolution for oxygenated species. Instead, FTIR analysis and adsorption tests were used to reliably identify and confirm the role of surface functionalities.

### 2.4. Adsorption of Dyes

The adsorption kinetics of dyes in aqueous solution were analyzed by mixing a specific amount of carbon with a fixed volume of dye solution of known concentration and agitating the mixture at a constant temperature. After preset contact time intervals, the solid and liquid phases were separated by filtration, and the dye concentration in the supernatant was determined. Adsorption was quantified by the difference between the initial and final concentrations due to solute retention by the adsorbent. Specifically, 0.1 g of adsorbent was weighed for all samples and placed in a series of 25 mL test tubes equipped with screw caps to prevent concentration changes due to solvent evaporation. Then, 20 mL of solutions of the dyes methylene blue (MB), methyl orange (MO), and orange G (OG), each at a concentration of 1.5 × 10^−3^ mol·L^−1^, were added. The tubes were placed in a thermostatic bath, Unitronic Orbital C, with water at 25 °C and an agitation speed of 50 oscillations/min. Each tube was left in the thermostatic bath for varying durations, from 15 min to 250 h. Initially, time intervals were short, increasing as the solution concentration stabilized, indicating adsorption/desorption equilibrium between the solute and the adsorbent. After each interval, phase contact was stopped, and the contents of the tubes were filtered. The absorbance of the resulting liquid was then measured using a Shimadzu UV-3101PC UV–Vis spectrophotometer with 1 cm optical path glass cuvettes, specifically at λ_max_ = 664 nm for MB, 464 nm for MO, and 477 nm for OG. The concentration data over time show how long it takes to reach adsorption/desorption equilibrium, key to determining the adsorption isotherm. All adsorption experiments were carried out in duplicate under the same experimental conditions, and the reported values correspond to the average of the replicates.

### 2.5. Computational Methods

The molecular structures of the dyes were optimized using Gaussian 16 software [40], employing the Density Functional Theory (DFT) with the M06-2X functional [41] and the 6-311++G(3df,3pd) basis set. To account for solvent effects, the SMD solvation model [42] was applied using water as the solvent. GaussView 6.0 software [43] was used for visualizing the optimized structures of the dyes, whose coordinates are provided in Tables S1–S3 (Supplementary Materials), and for depicting the molecular electrostatic potential (MEP). The van der Waals bounding boxes of the molecules were calculated using the Multiwfn 3.8 program [44,45], and the visualization was rendered using the VMD 1.9 tool [46].

## 3. Results and Discussion

### 3.1. Analysis of Starting Material

We first conducted an elemental analysis of the surgical masks (SM), and the results are presented in Table 1. The material exhibits high carbon and hydrogen contents, with low levels of sulfur and nitrogen. Of particular relevance to this study is the notably high carbon content and the absence of ash, which is advantageous for the preparation of carbonaceous adsorbent materials (ACMs). A low ash content in the resulting carbon helps prevent potential negative effects of inorganic residues on catalytic and adsorption processes, thereby enhancing the material’s applicability. These characteristics make SM a promising precursor for ACM production.

### 3.2. Analysis of Carbonaceous Adsorbent Materials: Chemical Treatment

When SM are heated in the temperature range between 25 and 900 °C, there is a significant mass loss of 98.73%. Based on these results, the possibility of preparing activated carbons from surgical masks by a traditional physical activation process, which would imply the loss of almost the entire sample, was ruled out. The preparation of the carbonaceous adsorbent materials was first carried out by the chemical activation method with sulfuric acid, and then with subsequent treatment of this sample by the physical activation method.

After the chemical activation of SM with H_2_SO_4_ as described in Materials and Methods, we obtained a yield value of 71.35%, suggesting that H_2_SO_4_ behaves as an oxidizing and dehydrating agent, and a carbonaceous residue with a high yield is obtained. Even so, loss of fine particles during washing and filtering is possible.

The elemental analysis data determined for carbonaceous adsorbent materials (ACM) is shown in Table 1. From the comparison of these results with those obtained for surgical masks, it can be deduced that, after the treatment with H_2_SO_4_, the carbon and hydrogen contents decrease while the oxygen and sulfur contents increase, the latter being what one would expect, which can be related to the formation of sulfonic groups on the surface of the charcoal. The ash content (1.67%, Table 1), although very low, increases slightly with respect to the starting material, surgical masks.

We performed a complete analysis of ACM using different techniques. First, we determined the total carbon (inorganic + organic) and found C. Inorg. = 1.914 mg·L^−1^ and C. Org. = 37.464 mg·L^−1^, and the results obtained by the total carbon method were found to be in agreement with those obtained by elemental analysis and immediate analysis.

#### 3.2.1. TG-DTG Analysis and Scanning Electron Microscopy

**Figure 1:**
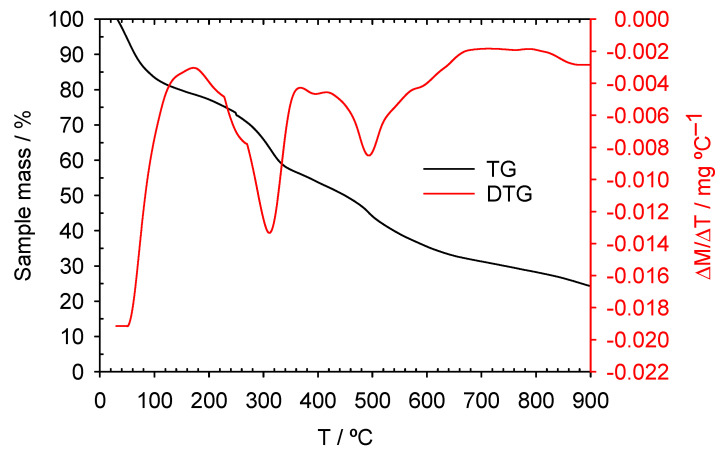
Thermogravimetric analysis. TG-DTG curve. Sample: carbonaceous adsorbent materials (ACM).

The TG-DTG curves for ACM, shown in Figure 1, indicate a steady mass loss of approximately 75% when the material is heated from room temperature to 900 °C under an inert atmosphere. This loss is consistent with the dehydration of the starting material during H_2_SO_4_ treatment. The DTG curve reveals a 30% mass loss below ~300 °C, attributed to the removal of retained water. At higher temperatures, an additional gradual mass loss of about 45% is observed, likely due to the devolatilization of organic components.

**Figure 2:**
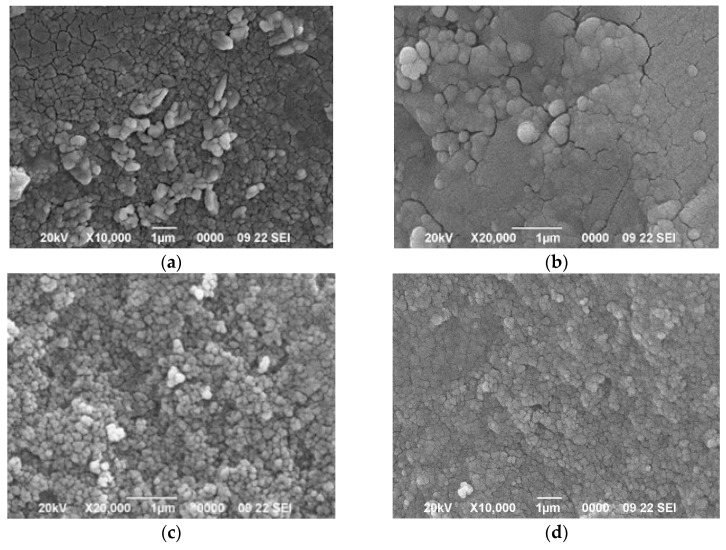
SEM micrographs of the different carbonaceous adsorbents: (**a**) ACM; (**b**) ACM-A; (**c**) ACM-CO_2_; (**d**) ACM-WV.

The scanning electron micrograph for the ACM sample can be seen in Figure 2a. The morphology shows a compact material surface with an absence of crevices and cavities and a rather earthy appearance.

#### 3.2.2. Textural Characterization

**Figure 3:**
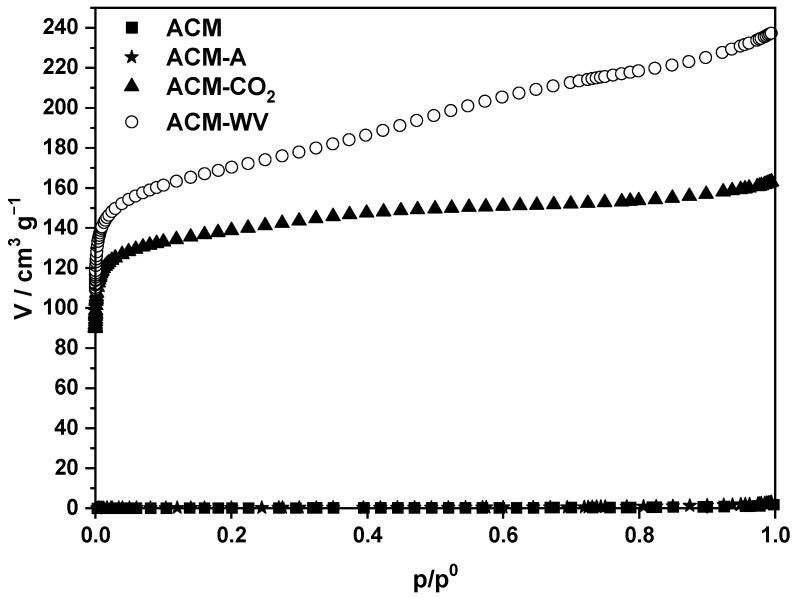
Adsorption isotherms of the different carbonaceous adsorbents.

The N_2_ adsorption isotherm at −196 °C measured for the ACM sample is shown in Figure 3. The low adsorption capacity of the sample is striking, which indicates that it has a poorly developed porous texture. On the other hand, the isotherm resembles type IV of the classification system of N_2_ adsorption isotherms at −196 °C proposed by BDDT [47]. This form of isotherm is characterized by a steeply sloping section at high *p*/*p*^0^ values, close to unity.

From the N_2_ adsorption isotherm at −196 °C, the values of S_BET_ (<1 m^2^·g^−1^), V_mi_ (0.0002 cm^3^·g^−1^), and V_me_ (0.0015 cm^3^·g^−1^) were calculated and corroborate the scarce development of porosity in ACM. The chemical treatment of SM does not produce porosity development in the material.

**Figure 4:**
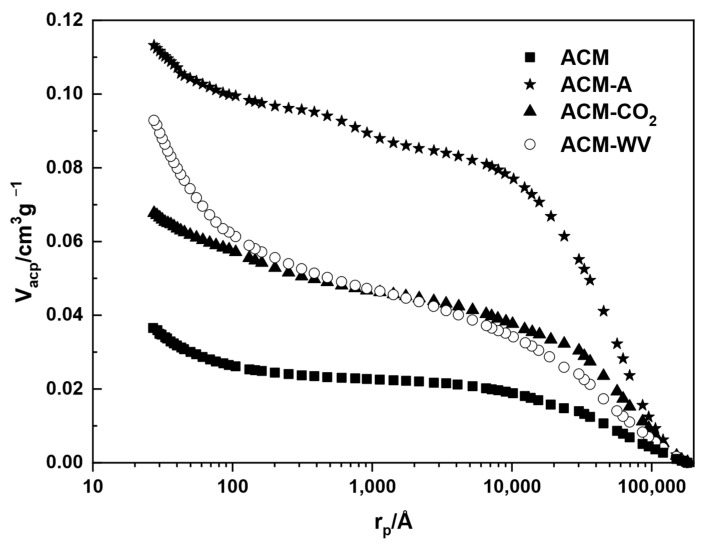
Mercury intrusion curves of the different carbonaceous adsorbents.

It is evident from the mercury intrusion curve obtained for ACM (Figure 4) that there is little development of both meso- and macro-porosity. It is inferred that macropores of different sizes and narrow mesopores predominate. The values of mesopore and macropore volumes, V_me-p_ (0.012 cm^3^·g^−1^) and V_ma-p_ (0.024 cm^3^·g^−1^), and mercury density (1.69 g·cm^−3^) suggest that when SM are treated with sulfuric acid, the porosity development is really low and therefore very ineffective.

#### 3.2.3. Chemical-Surface Characterization

**Figure 5:**
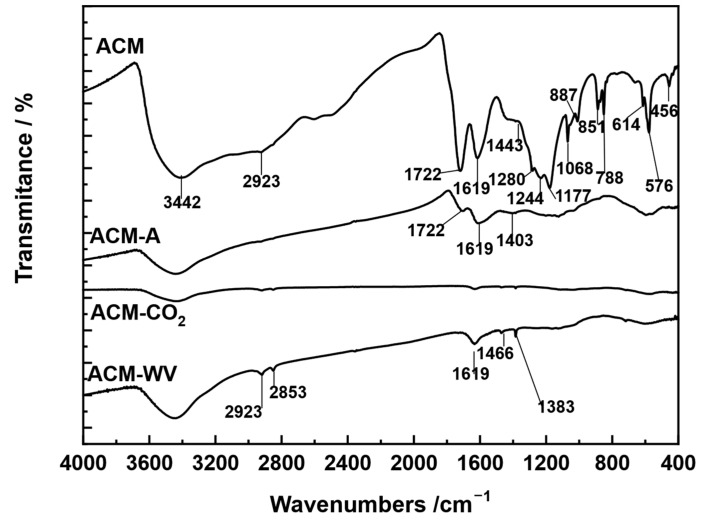
FT-IR spectra of the different carbonaceous adsorbents.

The FT-IR spectrum of ACM (Figure 5) shows the presence of a series of absorption bands located between 4000 and 400 cm^−1^ that can be assigned to the binding vibrations shown in Table S4 (Supplementary Materials). The intensity of the absorption bands at 3422, 2923, 1722, 1619, 1443, 1280, 1244, 1177, 1068, 788, 887, 851, 614, 576, and 456 cm^−1^ indicates that the most abundant functional groups in the ACM are hydroxyl groups, carboxylic acids, aromatic rings, methyl groups, and phenolic groups, as well as the presence of sulfonic groups. Nonetheless, it should be noted that the relative intensities of the O–H and C=O bands are influenced by the plotting scale of the spectra, and therefore, direct quantitative comparison between these bands should be considered with caution.

FT-IR spectroscopy can be used to determine functional groups in molecules anchored on a solid such as carbon. Since, in our case, we are dealing with a material that has a high carbon content (85.72% for SM) and has been treated with sulfuric acid, we identify sulfonated groups such as O=S=O and S=O. The structure of the sulfonated species anchored on the carbon surface could be attributed to the O-H vibration band at 3422 cm^−1^ for the ACM material. It is assumed that this bond is linked to the O=S-OH sulfonyl group resulting from the mild decomposition of sulfuric acid.

The measured pH_Sus_ of the ACM sample is notably low, with a value of 1.96, indicating a strongly acidic surface character. This low value corroborates the presence of acidic functional groups on the carbon surface. The pH_Sus_ is a critical parameter in interpreting adsorption behavior in solution, as it marks the pH at which the surface charge of the carbon transitions from positive to negative. Consequently, it influences the interaction between the adsorbent and ionic species in solution, helping to predict the pH range where adsorption will be more favorable, depending on whether the solute is cationic or anionic.

### 3.3. Physical Treatment

The yield values for the activation step of the overall process of preparing activated carbon by the physical activation method by air, CO_2_, and water vapor (respectively ACM-A, 45.60%; ACM-CO_2_, 33.14%; ACM-WV, 29.45%) show that the mass loss (=100 − yield), or burnup percentage, resulting from the partial gasification of carbon atoms of the carbonized products is not too high for when the activation is carried out in air. It is higher with CO_2_ and/or water vapor. The order of variation in the burning rate is ACM-WV > ACM-CO_2_ > ACM-A. This usually influences not only the porous texture of the activated carbon, but also the adsorption process of solutes in aqueous solution.

#### 3.3.1. Elemental Analysis

Elemental analysis data for samples ACM-A, ACM-CO_2_, and ACM-WV (Table 2) indicate that, as a consequence of activation, there is generally an increase in carbon content and a decrease in hydrogen and nitrogen content. It is worth noting the high oxygen content of sample ACM-A, perhaps due to the formation of oxygenated functional groups on the carbon surface.

As can also be seen in Table 2, the ash content of the activated samples is relatively low, even well below that of commercial activated carbons. Because of the activation processes, there is a progressive increase in inorganic matter both in the intermediate product (ACM) and in the final products. With regard to the possible applications of these activated carbons, as mentioned above, the ash content must be low (the limit is in the case of carbons used in medicine and pharmacy, which have a very low content of inorganic matter), and this content depends on the starting material.

#### 3.3.2. Scanning Electron Microscopy (SEM)

The scanning electron micrographs obtained for ACM-A, ACM-CO_2_, and ACM-WV (Figure 2b, Figure 2c, and Figure 2d, respectively) show that the activation of ACM leads to a porosity development that depends on the activating agent. The shape of the micrographs shows a compact surface, which clearly varies in the way of agglutination depending on the activating agent. The micrographs of the ACM-CO_2_ and ACM-WV samples are very similar to each other.

#### 3.3.3. Textural Characterization

The N_2_ isotherms obtained for ACM-A, ACM-CO_2_, and ACM-WV are also plotted in Figure 3 (see above), and textural data calculated from these isotherms are included in Table 3.

In view of both the isotherms and the aforementioned textural data, it is evident that physical activation of ACM is an effective method to prepare activated carbons with a high degree of surface area and porosity development, especially in the micropore region, although to a different extent depending on the activating agent, which also influences the porosity distribution of the activated carbon samples. In general, for a given activating agent, an increase in the burning rate promotes the creation of pores, which results in an increase in pore volume and specific surface area.

The activation atmosphere and temperature governed the textural evolution. At 400 °C (air), oxidation was mainly superficial, generating oxygenated groups with limited porosity development. At 800 °C (CO_2_), selective gasification favored micropore formation and a marked increase in S_BET_. Under 800 °C (steam), the stronger gasification expanded existing micropores into accessible mesopores, producing a hierarchical micro/mesoporous network and the highest surface areas. Consistently, the measured burn-off values (air ≈ 54.4%, CO_2_ ≈ 66.9%, steam ≈ 70.6%) parallel the increase in pore volumes and the adsorption capacities observed for dyes.

While the isotherms for ACM-A and ACM-CO_2_ are quite like the type I isotherm of the BDDT classification, the isotherm of ACM-WV is rather type IV. The latter isotherm could also be a composite isotherm of the type I and IV isotherms. The shape of the isotherms (see especially the opening of the bend and the evolution of N_2_ adsorption with increasing *p*/*p*^0^) is compatible with the presence of a wide distribution of porosity in the micropore zone and also of mesopores—but only narrow pores—of different sizes in ACM-CO_2_ and a very wide distribution of porosity in the micro- and mesopore regions in ACM-WV. For sample ACM-A, little porosity development is observed.

In short, from the N_2_ adsorption isotherms, it is inferred that the sample with the most heterogeneous porosity in the micro- and mesopore zones is ACM-WV. The fact that the isotherm of this sample over the whole range of w/w values does not show the presence of a turning point indicative of the onset of capillary condensation suggests that the mesopores present in this sample are sufficiently wide for adsorption to take place only by the well-known monolayer–multilayer mechanism. The relative position of the isotherms with respect to the ordinate axis indicates that the development of microporosity varies according to ACM-WV > ACM-CO_2_. In the case of mesoporosity, the order of variation is ACM-WV > ACM-CO_2_. As can be seen from Table 3, S_BET_ varies between 633 m^2^·g^−1^ for ACM-WV and 525 m^2^·g^−1^ for ACM-CO_2_.

The average pore width (APW) values reflect the differences in pore development among the studied carbons. For ACM and ACM-A, APW could not be determined due to their negligible surface area and pore volume, confirming the absence of a significant porous structure. In contrast, ACM-CO_2_ and ACM-WV exhibited well-developed porosity, with APW values of 28.5 Å and 37.8 Å, respectively. These mesopore dimensions are consistent with the enhanced surface areas (525 and 633 m^2^·g^−1^) and pore volumes (0.21 and 0.25 cm^3^·g^−1^), and they indicate that pore widening occurred more prominently under steam activation. The larger APW of ACM-WV, together with its higher pore volume, suggests improved accessibility for adsorbate molecules, which is likely to contribute to its superior adsorption performance.

To sum up, the activation atmosphere had a decisive impact on the pore structure and adsorption performance of the carbons. Air treatment mainly produced mild oxidation and surface functionalization, with limited porosity development. Activation with CO_2_ promoted the formation of micropores through selective gasification, resulting in higher surface areas. Steam activation, due to its stronger reactivity, further enlarged micropores into mesopores and generated a hierarchical porous structure with significantly higher surface area and pore volume. This explains the superior adsorption capacity of ACM-WV, which combines abundant micropores for adsorption sites with mesopores that facilitate dye diffusion.

The mercury intrusion curves obtained for ACM-A, ACM-CO_2_, and ACM-WV (Figure 4, see above) show that the porosity distribution is higher for ACM-A in the macropore zone than for the other two activated samples, being similar for ACM-CO_2_ and ACM-WV. For the mesopore zone, it is very similar for ACM-A and ACM-CO_2_. It is noteworthy that the presence of a narrow mesopore fraction for all samples is more pronounced for ACM-WV. Therefore, by activating in air, CO_2_, or a water vapor atmosphere, it is possible to prepare activated carbon with different pore sizes in the meso- and macropore regions from the masks. The generation of wide pores, such as macropores with O_2_ (N_2_) as activating agent, is usually related to fast kinetics of the gasification process, which leads to a shallow rather than a deep activation effect, as well as to the dilution of O_2_ by the N_2_ present in the gas stream. V_ma-p_ and V_me-p_ are high for all three samples, with V_ma-p_ being higher for ACM-A (Table 4). Table 4 compiles the measured apparent density (ρ_Hg_) values obtained for the samples. The density follows the order: ACM-A > ACM-CO_2_ > ACM-WV, which matches their porosity levels. Higher porosity means lower ρ_Hg_ because mercury does not enter the pores, making samples with more void space lighter and less dense.

#### 3.3.4. Chemical-Surface Characterization: FT-IR Spectroscopy and pH_Sus_

The FT-IR spectra recorded for ACM-A, ACM-CO_2_, and ACM-WV are also shown in Figure 5 (see above). These spectra show practically the same series of absorption bands, although with a different intensity. The presence of the same functional groups on the surface of the three activated carbon samples is compatible with the fact that the preparation of the activated carbon has always been carried out by an oxidation process of the carbonate, and, as a consequence, the tendency will always be to increase the oxidation state of the carbon through the formation of oxygenated structures. However, the organic functional group will be formed to a different extent depending more on kinetic factors than on thermodynamic factors. Thus, the oxidation progress with each of the three oxidizing agents during the activation treatment time will be the determining factor for the concentration of the groups ultimately present on the carbon surface. The highest intensity bands present in the spectra can be assigned to the following bond vibrational modes (in cm^−1^): 3422, υ(O-H); 2920 and 2853, υ(C-H); 1715, υ(C=O); 1623 and 1466, υ(C-C) skeletals; 1407 and 1383, δ(C-H); 1024, υ(C-O). As an example, it can be seen that the intensity of the band centered at 3442 cm^−1^ is more intense according to: ACM-WV > ACM-CO_2_ > ACM-A, which gives an idea of the relative presence of bonded (O-H) and sulfonyl groups in the samples.

Finally, as can be seen in Table 5, the pH_Sus_ is higher than 7.0 for ACM-CO_2_ and ACM-WV and slightly lower than 7.0 for ACM-A. The basic character of the first two carbons in such a wide pH range may be due to the presence of C=O groups in pyrone-type structures, electrons in the graphene layers, inorganic impurities, etc. When interpreting the pH_Sus_ values, it should be taken into account that this pH is the result of weighing the acidic groups against the basic groups present on the surface of each carbon and that, according to the volumetric titration method used to measure it, it is a function not only of the content of one group or the other, but also of the strength of these groups as acids or bases. It should be noted that ACM-CO_2_ and ACM-WV, which are the samples with the highest pH_Sus_, are also those with the highest content of structures with C=O bonds.

### 3.4. Adsorption of Dyes

**Figure 6:**
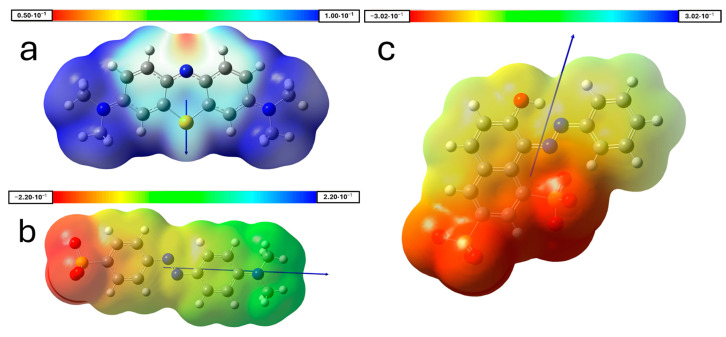
Molecular electrostatic potential (MEP) at an isovalue of 0.0004 a.u., and dipole moment (Debyes) of (**a**) Methylene blue cation, visualized on a positive charge gradient, 3.2 D; (**b**) Methyl orange anion, 38.2 D; (**c**) Orange G dianion, 29.6 D.

In this work, we investigated the properties of carbonaceous adsorbent materials prepared from surgical masks on the adsorption of the dyes methylene blue (MB), methyl orange (MO), and orange G (OG) in aqueous solution. It is important to note that these dyes are salts that, in solution, will give rise to ions. In the case of MB, it will be a cation; MO will be an anion; and OG will be a dianion. The molecular electrostatic potential (MEP), which describes the charge distribution in these ions, can be seen in Figure 6. In this way, the ions can interact with the surface of the adsorbent, whose net charge will be defined by its pH_Sus_.

**Figure 7:**
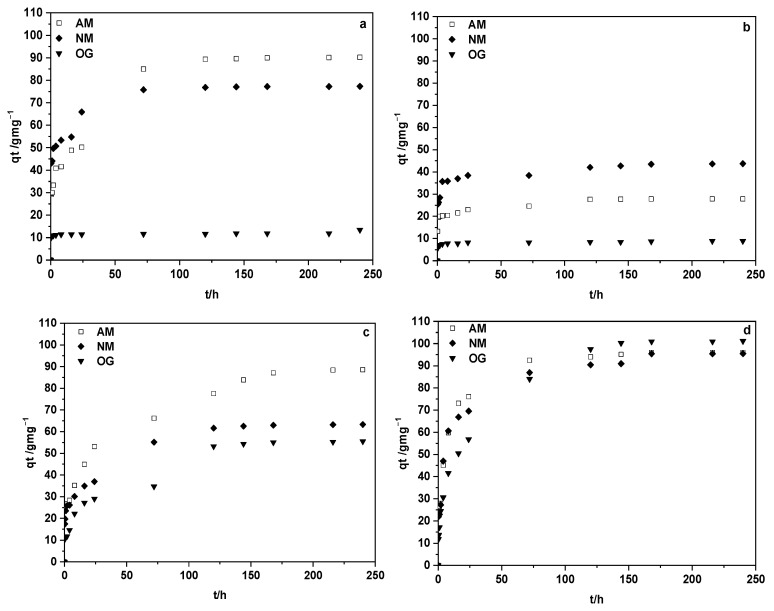
Kinetics of dye adsorption (methylene blue, MB; methyl orange, MO; and orange G, OG) by different samples: (**a**) ACM; (**b**) ACM-A; (**c**) ACM-CO_2_; (**d**) ACM-WV.

The adsorption kinetics, plotted as *q*_t_ vs. t for all the adsorption systems formed for each of the carbonaceous adsorbent material samples and the dyes under study, dissolved in Milli-Q water at an initial concentration of 1.5 × 10^−3^ M, are given in Figure 7.

The adsorption kinetic data were fitted to two commonly used models, namely, Pseudo-first order (PFO) and Pseudo-second order (PSO). The PFO model, first proposed by Lagergren [48], assumes that the adsorption rate is proportional to the number of unoccupied sites, following the differential form

(2) $$\frac{dq_{t}}{dt}=k_{1}\left(q_{e}-q_{t}\right)$$

Its linear expression is

(3) $$ln\left(q_{e}-q_{t}\right)=\ln q_{e}-k_{1}t$$

where *k*_1_ (h^−1^) is the rate constant, and *q*_t_ and *q*_e_ are the adsorption capacities at time *t* and equilibrium, respectively. This model typically describes the initial, rapid adsorption stages dominated by physisorption.

The PSO model, developed by Ho and McKay [49], assumes that the rate-limiting step is proportional to the square of the number of unoccupied sites, usually consistent with chemisorption processes. It is expressed as

(4) $$\frac{dq_{t}}{dt}=k_{2}{\left(q_{e}-q_{t}\right)}^{2}$$

with its linearized form given by

(5) $$\frac{t}{q_{t}}=\frac{1}{k_{2}q_{e}^{2}}+\frac{t}{q_{e}}$$

where *k*_2_ (g·mg^−1^·h^−1^) is the rate constant. The PSO model generally provides a better fit for adsorption of dyes and other organics onto activated carbons, often yielding correlation coefficients (*R*^2^) close to unity and equilibrium adsorption capacities in good agreement with experimental values.

In this study, both models were applied, and the most suitable description of the kinetic data was identified based on the goodness of fit (*R*^2^) and the consistency between calculated and experimental values.

Table 6 shows kinetics and equilibrium data calculated according to the PFO and PSO models. For completeness, the plots illustrating the fit of the experimental data to these models are presented in the Supplementary Material (Figures S1 and S2). Although both models provided relatively high correlation coefficients (*R*^2^ > 0.95 in most cases), the PSO model consistently yielded theoretical adsorption capacities (*q*_e_) that were much closer to the experimental values. This indicates that the PSO model better describes the adsorption process, not only in terms of statistical fit but also in predictive reliability. The agreement between experimental and calculated *q*_e_ values suggests that adsorption is governed predominantly by chemisorption processes, involving specific interactions between dye molecules and surface functional groups of the activated carbons.

The value of *q*_e_, as expected, is notably higher for the ACM-WV system for the three dyes. High *q*_e_ values are also obtained for the systems formed with ACM and MB and MO.

The C = f(t) curves for the products resulting from the chemical and physical treatments show that the variation in concentration with time is very small for the ACM-A systems and the three dyes than for most of the adsorption systems studied. In the case of the ACM-A and ACM-WV adsorbents, it seems that the adsorption process, from the point of view of its kinetics, takes place in two stages: a first stage with fast kinetics and a subsequent one with slower kinetics. This behavior may be attributed to differences in the accessibility of active sites on the sorbent surface—where readily accessible external sites are occupied rapidly in the initial phase, followed by slower diffusion and occupation of less accessible internal or microporous sites during the second phase. Undoubtedly, the kinetics of the process in its first stage are faster for MB and MO for the two adsorbents ACM-A and ACM-WV (Figure 7).

Based on the results obtained for the equilibrium time and *q*_e_ for the different adsorbate/adsorbent systems studied, the possible application of the samples prepared in this work for the retention of the dyes used under study in real samples, specifically water from the Guadiana River, is investigated. Previously, filtered river water samples were used, and the dyes under study were added in concentrations of the order of 1.5 × 10^−3^ M. The analysis is carried out in triplicate. Table 7 shows the dye retention values for each of the adsorbents prepared. In view of the results obtained, we can say that ACMs have been prepared from surgical masks with a high applicability for the removal of dyes in real samples. Data obtained with ultrapure water as a control confirm these results.

Interpreting adsorption results can sometimes be a complex task, as they depend on numerous factors. These are mainly related to the textural properties of the adsorbents and to the size and shape of the adsorbates. Another relevant aspect is that the adsorbates are ionic chemical species, which in solution bear positive charges (MB) or negative charges (MO and OG; in the latter case, doubly charged), and can thus engage in electrostatic interactions with functional groups on the adsorbent surface. These charges also influence the orientation adopted by the adsorbate on the surface. Regarding this orientation factor, the polarity of the adsorbate should also be considered (see Figure 6). A global descriptor of adsorbate polarity is the topological polar surface area (TPSA), defined as the sum of the surface areas of the polar atoms in a molecule (especially O and N, as well as H atoms bonded to them with the ability to form hydrogen bonds) [50,51]. The TPSA is 43.9 Å^2^ for MB, 93.5 Å^2^ for MO, and 176 Å^2^ for OG [52]. This is also important because, in aqueous solution, the dye ions can become hydrated, potentially resulting in an effective adsorbate area that is larger than expected. In such cases, the overall charge becomes delocalized over a greater number of atoms, which may act as a limiting factor for the diffusion of the adsorbate into the pores of the adsorbent. As a result, adsorption becomes more dependent on the adsorbate’s ability to form π bonds.

**Figure 8:**
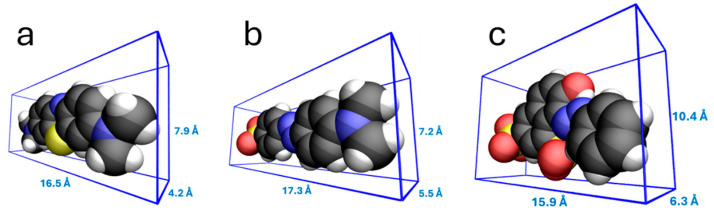
Van der Waals bounding boxes of (**a**) Methylene blue cation; (**b**) Methyl orange anion; (**c**) Orange G dianion. Colors represent C (black), H (white), N (blue), O (red), and S (yellow).

The data indicate that, for all the studied dyes, the ACM-WV sample is the most efficient adsorbent. This is consistent because it exhibits the highest volumes of micropores and mesopores, supported by a high surface area and a type IV isotherm, suggesting accessible mesopores. In the cationic methylene blue, the adsorption was elevated despite the positive charge of the carbon surface (pH_Sus_ = 9.25). This could be justified by the presence of oxygenated groups (6.96% O, e.g., -OH, -CO) capable of forming hydrogen bonds alongside π–π interactions, together counteracting electrostatic repulsions. Although the dianion of Orange G interacts with the net positive surface, its retention was lower in ultrapure water, likely due to its larger size (see Figure 8), which limits access to micropores. However, when using river water, the retention of Orange G increased. This could be attributed to the incorporation of ionic bridges formed by divalent cations (e.g., Ca^2+^ and Mg^2+^) between residual oxygenated groups and the dye [53].

The ACM-CO_2_ sample exhibits a micropore volume similar to that of the previous sample and a type I isotherm, which corresponds to a predominantly microporous structure. However, the mesopore volume is significantly lower, and since the dye must reach the internal micropores through these mesopores, a lower extent of adsorption would be expected, as is indeed the case. Additionally, the mesopore volume determined from the N_2_ adsorption isotherm is greater than that obtained from mercury porosimetry, which is usually indicative of mercury being unable to access all mesopores, likely due to bottlenecks or microporous necks. This would support the observation that larger molecules such as OG are less retained. In river water, the presence of salts and dissolved organic matter may reduce dye adsorption by blocking microporous channels and preventing access to active sites. On the other hand, the positively charged surface of the material may promote the adsorption of anions such as MO and OG at the entrances of microporous channels, forming steric ‘plugs’ that would reduce global efficiency.

The ACM and ACM-A samples show very low micro- and mesoporosity, which is generally associated with a reduced adsorption capacity. Nevertheless, an exception can be observed in the high retention of MB, likely due to the negatively charged surface of the adsorbent. Methylene blue, upon dissociation, produces a cation with a delocalized positive charge across the molecule, making it prone to electrostatic interactions with the carbon surface (especially with sulfonic groups, which are dissociated and negatively charged at neutral pH).

To better assess the innovativeness of this work, it is worth comparing the adsorption performance of mask-derived carbons with values reported for commercial activated carbons and other biomass-based adsorbents. Typical commercial ACs show methylene blue adsorption capacities in the range of 200–400 mg·g^−1^, while biomass-derived carbons usually achieve 150–500 mg·g^−1^ depending on activation conditions [54,55,56]. In our case, the steam-activated material (ACM-WV) reached nearly complete removal of MB and >95% removal of MO under the tested conditions, which are fully comparable with or superior to many commercial references.

To benchmark the performance of the mask-derived activated carbons here reported against state-of-the-art adsorbents, Table 8 compiles recent (2020–2025) reports on methylene blue (MB), methyl orange (MO), and related azo dyes using commercial activated carbons (CACs) and comparable biomass-derived carbons. Under the conditions applied here, ACM-WV shows equilibrium uptakes of ~98 mg·g^−1^ for MB and MO and ~110 mg·g^−1^ for Orange G (values converted from pseudo-second-order *q*_e_ in Table 6), which place our material within the range of practical CACs and many low-to-mid capacity biomass carbons, despite using a waste-mask precursor and simple activation. Higher capacities reported in the literature typically stem from aggressive chemical activation, composite formation, or narrow test conditions (e.g., very low adsorbent dose, extreme pH), which are less directly comparable to our application-focused protocol.

## 4. Conclusions

From the results obtained in this study, the following conclusions may be drawn:

- Disposable surgical masks, largely composed of polypropylene, represent a growing source of microplastic pollution but also a promising carbon-rich precursor.
- Chemical treatment with H_2_SO_4_ followed by physical activation enabled the preparation of activated carbons (ACMs) with enhanced surface chemistry and porosity.
- Steam activation (ACM-WV) yielded the highest surface area and adsorption capacity, achieving nearly complete removal of methylene blue and high efficiencies for methyl orange and orange G.
- Adsorption followed pseudo-second-order kinetics and was strongly influenced by pore structure, surface functional groups, and dye molecular properties.
- The ACMs proved effective not only in ultrapure water but also in natural river water, confirming their potential for real-world wastewater treatment.
- Future work will address scaling up the process, extending adsorption studies to a wider range of emerging contaminants (pharmaceuticals, pesticides, heavy metals), and assessing regeneration and reuse for industrial applications.

## Figures and Tables

**Table 1:** Elemental and immediate analysis data (% by weight). Samples: surgical masks (SM) and carbonaceous adsorbent material (ACM).

Sample	C	H	N	S	O	Ashes
SM	85.72	14.76	0.00	0.90	1.87	0.00
ACM	40.10	2.26	0.00	6.09	47.90	1.67

**Table 2:** Elemental and immediate analysis data (% by weight). Samples: ACM, ACM-A, ACM-CO_2_, and ACM-WV.

Sample	C	H	N	S	O	Ashes
ACM	40.10	2.26	0.00	6.09	47.90	1.67
ACM-A	58.29	1.82	0.00	1.89	30.15	3.42
ACM-CO_2_	78.93	0.72	0.00	1.22	3.37	5.74
ACM-WV	79.36	1.03	0.00	1.41	6.96	5.88

**Table 3:** N_2_ adsorption isotherms at −196 °C. Textural data. Samples: ACM-A, ACM-CO_2_, and ACM-WV.

Sample	S_BET_/m^2^·g^−1^	W_0_/cm^3^·g^−1^	V_mi_/cm^3^·g^−1^	V_me_/cm^3^·g^−1^	APW/Å
ACM	<1	0.00	0.00	0.00	n.a.
ACM-A	1	0.00	0.00	0.01	n.a.
ACM-CO_2_	525	0.21	0.21	0.04	28.5
ACM-WV	633	0.25	0.25	0.11	37.8

Key: APW: Average pore width; n.a.: not available.

**Table 4:** Mercury porosimetry. Volumes of meso- and macropores, and apparent density. Samples: ACM, ACM-A, ACM-CO_2_, and ACM-WV.

Sample	V_me-p_ (cm^3^·g^−1^)	V_ma-p_ (cm^3^·g^−1^)	ρ_Hg_ (g·cm^−3^)
ACM	0.012	0.024	1.690
ACM-A	0.017	0.096	1.421
ACM-CO_2_	0.015	0.052	1.310
ACM-WV	0.039	0.054	1.182

**Table 5:** pH_Sus_ values. Samples: ACM, ACM-A, ACM-CO_2_, and ACM-WV.

Sample	pH_Sus_	Surface Net Charge at pH 7
ACM	1.96	Negative
ACM-A	5.25	Negative
ACM-CO_2_	8.25	Positive
ACM-WV	9.25	Positive

**Table 6:** Kinetic and equilibrium data of dye adsorption (methylene blue, MB; methyl orange, MO; and orange G, OG) by different samples of carbonaceous adsorbent materials.

				Pseudo-First-Order Kinetic Model			Pseudo-Second-Order Kinetic Model		
Material	Sample	t_e_ (h)	*q*_e_ (Experimental mg·g^−1^)	*q*_e_ (mg·g^−1^)	*k*_1_ (h^−1^)	*R* ^2^	*q*_e_ (mg·g^−1^)	*k*_2_ (g·mg^−1^ ·h^−1^)	*R* ^2^
ACM	MB	100	90.18	65.43	0.0355	0.9899	92.93	0.0016	0.9963
	MO	100	77.32	30.88	0.0338	0.9904	78.08	0.0055	0.9996
	OG	120	13.45	2.13	0.0016	0.8853	11.85	0.1878	0.9999
ACM-A	MB	120	27.88	10.30	0.0302	0.9654	28.10	0.0139	0.9991
	MO	130	43.72	13.67	0.0207	0.9423	43.73	0.0103	0.9991
	OG	6	8.89	1.34	0.0092	0.9511	8.81	0.0677	0.9991
ACM-CO_2_	MB	200	88.55	65.53	0.0192	0.9547	90.32	0.0011	0.9924
	MO	130	63.31	44.06	0.0273	0.9969	64.96	0.0022	0.9967
	OG	130	55.46	48.73	0.0253	0.9704	57.68	0.0018	0.9955
ACM-WV	MB	130	95.93	55.60	0.0302	0.9607	97.58	0.0026	0.9997
	MO	180	95.45	57.35	0.0226	0.9156	96.71	0.0021	0.9989
	OG	150	101.12	85.32	0.0285	0.9757	105.28	0.0010	0.9961

**Table 7:** Dye retention. Samples: ACM, ACM-A, ACM-CO_2_, and ACM-WV.

Retention (%) (Ultrapure Water)				Retention (%) (River Water)		
Sample	Methylene Blue	Methyl Orange	Orange G	Methylene Blue	Methyl Orange	Orange G
ACM	94	79	10	91	83	12
ACM-A	29	45	5	26	42	4
ACM-CO_2_	92	64	41	59	60	21
ACM-WV	100	97	74	100	97	94

**Table 8:** Recent adsorption performance of commercial and biomass-derived carbons vs. this work (2020–2025).

Adsorbent (Type/Precursor)	Dye(s)	Performance (Capacity/Removal)	Key Notes	Reference
ACM-WV (this work)	MB, MO, Orange G	MB ≈ 98 mg·g^−1^; MO ≈ 98 mg·g^−1^; OG ≈ 110 mg·g^−1^	-	This work
Commercial AC (powder)	MO	q_max_ = 129.8 mg·g^−1^; removal 97.8%	Batch; Langmuir; pH ≈ 3	[57]
Commercial AC (fixed-bed)	MO	q ≈ 16.9 mg·g^−1^	Continuous column	[58]
Coconut shell AC	MB	q_max_ = 30.3 mg·g^−1^	Batch; Langmuir; pH ~6	[59]
Caraway seed AC (K_2_CO_3_)	MB, MR	MB = 296 mg·g^−1^; MR = 203 mg·g^−1^	Batch	[60]
Wood sawdust AC	MO	31.93 mg·g^−1^	pH 3; 60 min	[61]
Wood sawdust ZnO@AC	MO	42.61 mg·g^−1^	Improved vs. AC	[61]
Mesoporous AC	MB	≈1000 mg·g^−1^	Very high SSA	[62]
Microporous KOH-AC	MB	136.5 mg·g^−1^	Micropore-rich	[63]
Water-vapor AC	MB	148.8 mg·g^−1^	Fast removal	[64]
Pepper-stem AC	MB	≈75 mg·g^−1^	RSM optimized	[65]
Nutmeg shell AC/K_2_CO_3_	MB	346.9 mg·g^−1^	High porosity	[66]
Surfactant-modified AC (SLS-C)	MB	Virgin AC = 153.8; SLS-C = 232.5 mg·g^−1^	Surfactant effect	[67]
Recycled epoxy-board AC	MO	23.1–37.2 mg·g^−1^	Temp-dependent	[68]
Date-palm ZnO@DPS-AC	MO	227 mg·g^−1^	Rapid uptake	[69]
Magnetic AC (MAC)	MO	≈101 to 108 mg·g^−1^	Langmuir fits	[70]
Nanoporous carbon (ZnCl_2_)	MO	367.8 mg·g^−1^	Recyclable	[71]
Biosolids/cardboard KOH-AC	MB	≈191 mg·g^−1^	Batch	[72]
Sugarcane AC/zeolite	MB	≈51 mg·g^−1^	Composite	[73]
Golden-needle mushroom AC/KOH	MB, MO	MB = 816 mg·g^−1^; MO = 287 mg·g^−1^	Biomass self-activation	[74]
Porous carbon (PC-900)/KOH	MB, MO	MB = 1853.6 mg·g^−1^; MO = 927 mg·g^−1^	Very high capacity	[75]

## Data Availability

The original contributions presented in this study are included in the article. Further inquiries can be directed to the corresponding author.

## Supplementary Materials

The following supporting information can be downloaded at: https://www.mdpi.com/article/10.3390/ma18174115/s1. Figure S1: Pseudo-first-order plots for the adsorption of the three dyes by the carbonaceous sorbents; Figure S2: Pseudo-second-order plots for the adsorption of the three dyes by the carbonaceous sorbents; Table S1: Cartesian coordinates in pdb format for the optimized structure of methylene blue cation at the M06-2X/6-311++G(3df,3pd) level, with the SMD solvation model (solvent: water); Table S2: Cartesian coordinates in pdb format for the optimized structure of methyl orange anion at the M06-2X/6-311++G(3df,3pd) level, with the SMD solvation model (solvent: water); Table S3: Cartesian coordinates in pdb format for the optimized structure of orange G dianion at the M06-2X/6-311++G(3df,3pd) level, with the SMD solvation model (solvent: water); Table S4: FT-IR spectrum. Band assignment. Sample: carbonaceous adsorbent material (ACM).

## Abbreviations

The following abbreviations are used in this manuscript:

AC	Activated carbon
ACM	Carbonaceous adsorbent material
ACM-A	Air-activated carbon sample
ACM-CO_2_	CO_2_-activated carbon sample
ACM-WV	Water vapor-activated carbon sample
APW	Average pore width
ASPT	Área de la superficie polar topológica
BDDT	Brunauer, Deming, Deming, and Teller
ρ_Hg_	Apparent density of the sample
DSC	Differential Scanning Calorimetry
*p*/*p*^0^	Relative pressure
pH_Sus_	pH at the point of the aqueous suspension
S_BET_	Specific surface area
SCAI-UMA	Research Support Center of the University of Málaga
SEM	Scanning Electron Microscopy
SM	Surgical mask
TG	Thermogravimetry
TPSA	Topological polar surface area
V_ma-p_	Macropore volume (mercury porosimetry)
V_me-p_	Mesopore volume (mercury porosimetry)
V_me_	Mesopore volume (N_2_ adsorption isotherm)
V_mi_	Micropore volume (N_2_ adsorption isotherm)
