# Supplementary material for: Waste Surgical Masks as Precursors of Activated Carbon: A Circular Economy Approach to Mitigate the Impact of Microplastics and Emerging Dye Contaminants
Source: Materials (Basel). 2025 Sep 2;18(17):4115. doi: 10.3390/ma18174115 (PMC12429826; doi:10.3390/ma18174115)
Supplement: Supplementary file 1 [file materials-18-04115-s001.zip › materials-3823376-supplementary.pdf]

Supplementary Materials

for

Waste Surgical Masks as Precursors of Activated Carbon: A Circular Economy Approach to Mitigate the Impact of Microplastics and Emerging Dye Contaminants

María del Mar García-Galán [1], Carlos A. Fernández-Blanco [2], Eduardo M. Cuerda-Correa [2], Juan M. Garrido-Zoido [2,*] and María F. Alexandre-Franco [2,*]

[1] Departamento de Dirección de Empresas y Sociología, Universidad de Extremadura, Avenida de Elvas s/n, 06006 Badajoz, Spain; margalan@unex.es (M.d.M.G.-G.)

[2] Departamento de Química Orgánica e Inorgánica, Facultad de Ciencias, Universidad de Extremadura, Avenida de Elvas s/n, 06006 Badajoz, Spain; cfernandaf@alumnos.unex.es (C.A.F.-B.); emcc@unex.es (E.M.C.-C.)

* Correspondence: jmgarridoz@unex.es (J.M.G.-Z.); malexandre@unex.es (M.F.A.-F.); Tel.: +34-924486964 (M.F.A.-F.)

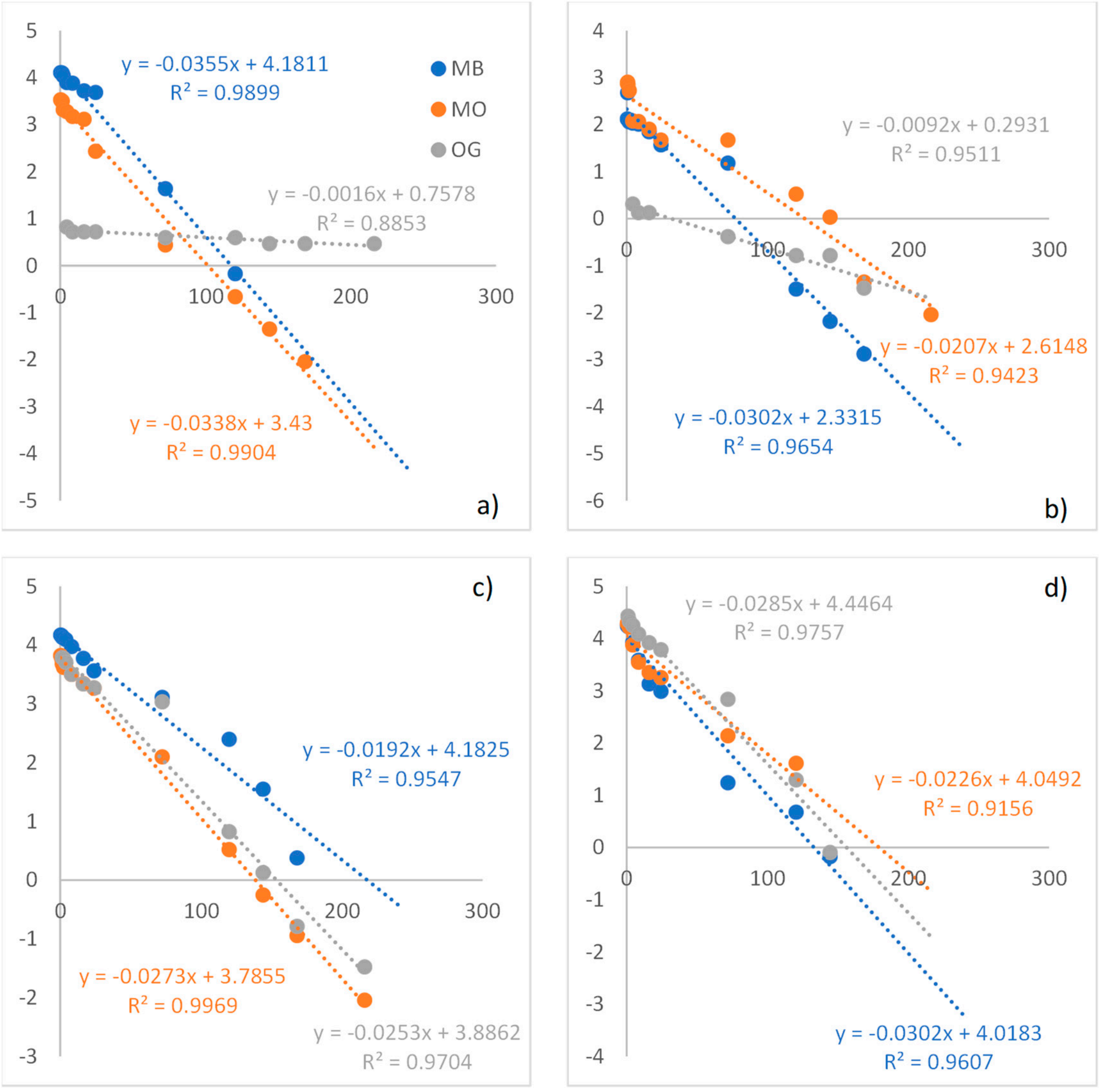

Figure S1. *Pseudo-first order plots (ln(q_e-q_t) vs t) for the adsorption of the three dyes by the carbonaceous sorbents. a) ACM; b) ACM-A; c) ACM-CO2; d) ACM-WV.*

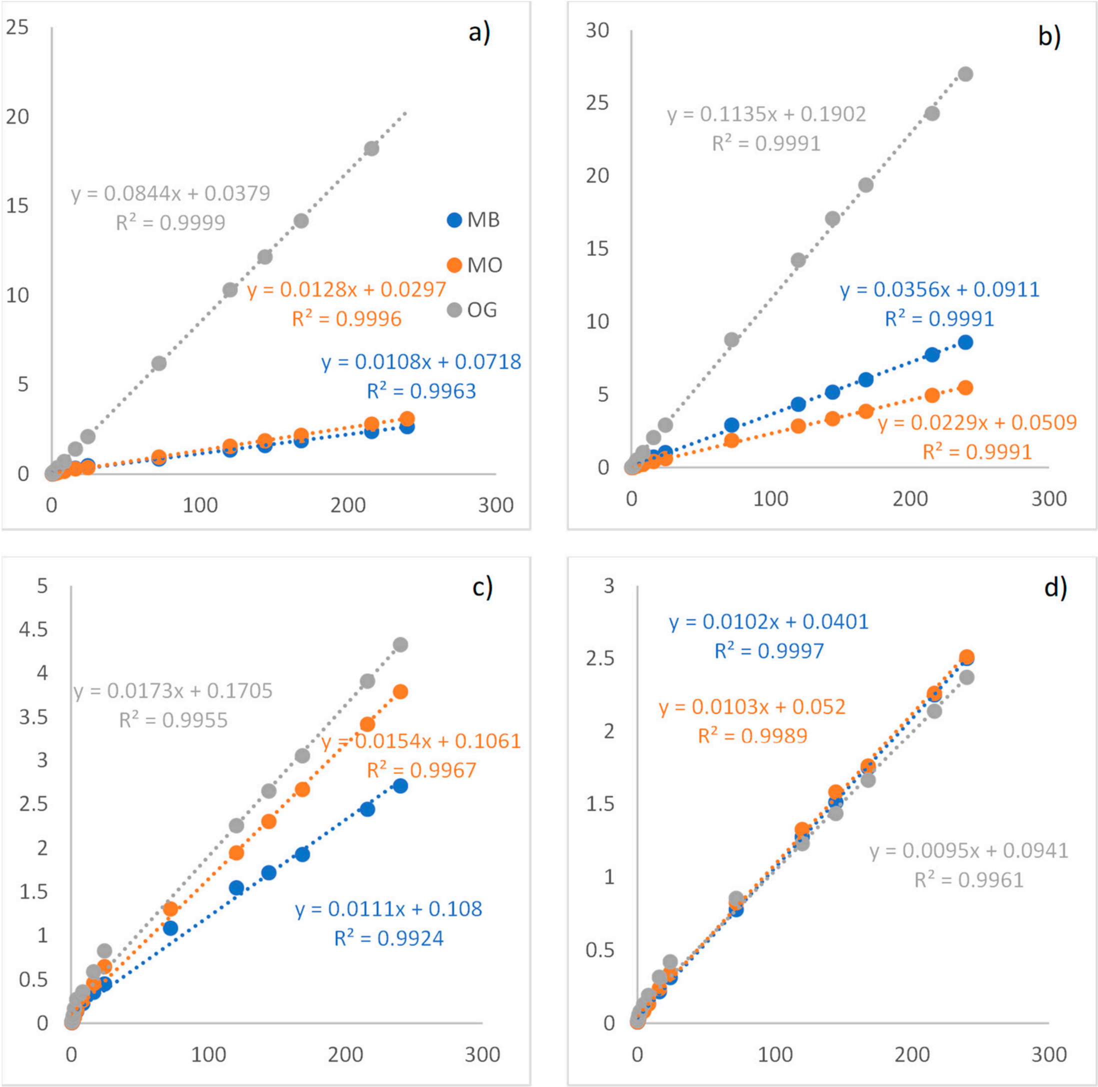

Figure S2. *Pseudo-second order plots (t/q_t vs t) for the adsorption of the three dyes by the carbonaceous sorbents. a) ACM; b) ACM-A; c) ACM-CO2; d) ACM-WV.*

***Table S1**. Cartesian coordinates in pdb format for the optimized structure of methylene blue cation at the M06-2X/6-311++G(3df,3pd) level, with the SMD solvation model (solvent: water)*

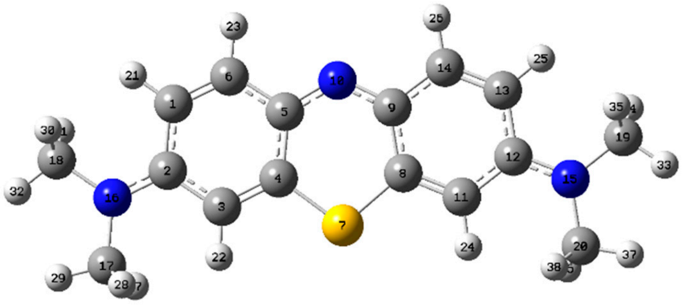

HETATM	1	C	0	-3.590	1.419	-0.000	C
HETATM	2	C	0	-3.768	-0.007	-0.000	C
HETATM	3	C	0	-2.614	-0.828	-0.000	C
HETATM	4	C	0	-1.359	-0.268	-0.000	C
HETATM	5	C	0	-1.171	1.152	-0.000	C
HETATM	6	C	0	-2.349	1.956	-0.000	C
HETATM	7	S	0	-0.000	-1.334	-0.000	S
HETATM	8	C	0	1.359	-0.268	-0.000	C
HETATM	9	C	0	1.171	1.152	-0.000	C
HETATM	10	N	0	0.000	1.775	-0.000	N
HETATM	11	C	0	2.614	-0.828	-0.000	C
HETATM	12	C	0	3.768	-0.007	-0.000	C
HETATM	13	C	0	3.590	1.419	-0.000	C
HETATM	14	C	0	2.349	1.956	-0.000	C
HETATM	15	N	0	4.992	-0.538	-0.000	N
HETATM	16	N	0	-4.992	-0.538	-0.000	N
HETATM	17	C	0	-5.168	-1.985	0.000	C
HETATM	18	C	0	-6.177	0.315	0.000	C
HETATM	19	C	0	6.177	0.315	0.000	C
HETATM	20	C	0	5.168	-1.985	0.000	C
HETATM	21	H	0	-4.452	2.067	0.000	H
HETATM	22	H	0	-2.714	-1.902	-0.000	H
HETATM	23	H	0	-2.213	3.029	0.000	H
HETATM	24	H	0	2.714	-1.902	-0.000	H
HETATM	25	H	0	4.452	2.067	0.000	H
HETATM	26	H	0	2.213	3.029	-0.000	H
HETATM	27	H	0	-4.715	-2.430	-0.886	H
HETATM	28	H	0	-4.715	-2.430	0.886	H
HETATM	29	H	0	-6.228	-2.208	0.000	H
HETATM	30	H	0	-6.201	0.947	0.886	H
HETATM	31	H	0	-6.201	0.948	-0.886	H
HETATM	32	H	0	-7.056	-0.317	-0.000	H
HETATM	33	H	0	7.056	-0.317	0.000	H
HETATM	34	H	0	6.201	0.948	-0.886	H
HETATM	35	H	0	6.201	0.947	0.887	H
HETATM	36	H	0	4.715	-2.430	-0.886	H
HETATM	37	H	0	6.228	-2.208	-0.000	H
HETATM	38	H	0	4.715	-2.430	0.886	H

END

***Table S2**. Cartesian coordinates in pdb format for the optimized structure of methyl orange anion at the M06-2X/6-311++G(3df,3pd) level, with the SMD solvation model (solvent: water)*

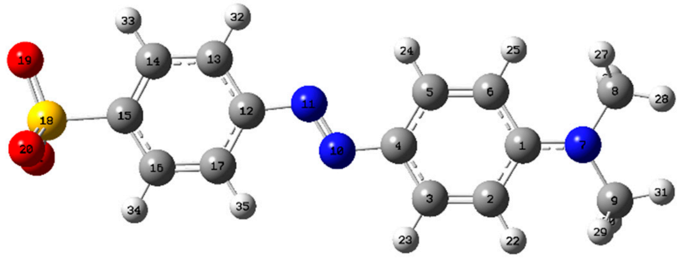

HETATM	1	C	0	5.179	-0.050	0.086	C
HETATM	2	C	0	4.525	-1.301	0.058	C
HETATM	3	C	0	3.149	-1.372	0.043	C
HETATM	4	C	0	2.362	-0.221	0.045	C
HETATM	5	C	0	2.997	1.026	0.053	C
HETATM	6	C	0	4.369	1.113	0.068	C
HETATM	7	N	0	6.542	0.039	0.138	N
HETATM	8	C	0	7.174	1.317	-0.152	C
HETATM	9	C	0	7.327	-1.155	-0.134	C
HETATM	10	N	0	0.972	-0.421	0.028	N
HETATM	11	N	0	0.263	0.601	0.038	N
HETATM	12	C	0	-1.138	0.355	0.022	C
HETATM	13	C	0	-1.948	1.484	0.018	C
HETATM	14	C	0	-3.331	1.356	0.003	C
HETATM	15	C	0	-3.895	0.092	-0.007	C
HETATM	16	C	0	-3.089	-1.045	-0.003	C
HETATM	17	C	0	-1.715	-0.918	0.012	C
HETATM	18	S	0	-5.660	-0.127	-0.024	S
HETATM	19	O	0	-6.242	1.207	-0.033	O
HETATM	20	O	0	-5.970	-0.880	1.187	O
HETATM	21	O	0	-5.946	-0.886	-1.238	O
HETATM	22	H	0	5.096	-2.215	0.047	H
HETATM	23	H	0	2.658	-2.336	0.024	H
HETATM	24	H	0	2.406	1.930	0.042	H
HETATM	25	H	0	4.825	2.090	0.066	H
HETATM	26	H	0	6.926	1.677	-1.155	H
HETATM	27	H	0	6.873	2.073	0.571	H
HETATM	28	H	0	8.249	1.198	-0.079	H
HETATM	29	H	0	7.114	-1.935	0.595	H
HETATM	30	H	0	7.132	-1.554	-1.134	H
HETATM	31	H	0	8.380	-0.906	-0.055	H
HETATM	32	H	0	-1.486	2.461	0.027	H
HETATM	33	H	0	-3.962	2.233	0.000	H
HETATM	34	H	0	-3.540	-2.028	-0.010	H
HETATM	35	H	0	-1.087	-1.796	0.016	H
END							

***Table S3**. Cartesian coordinates in pdb format for the optimized structure of orange G dianion at the M06-2X/6-311++G(3df,3pd) level, with the SMD solvation model (solvent: water)*

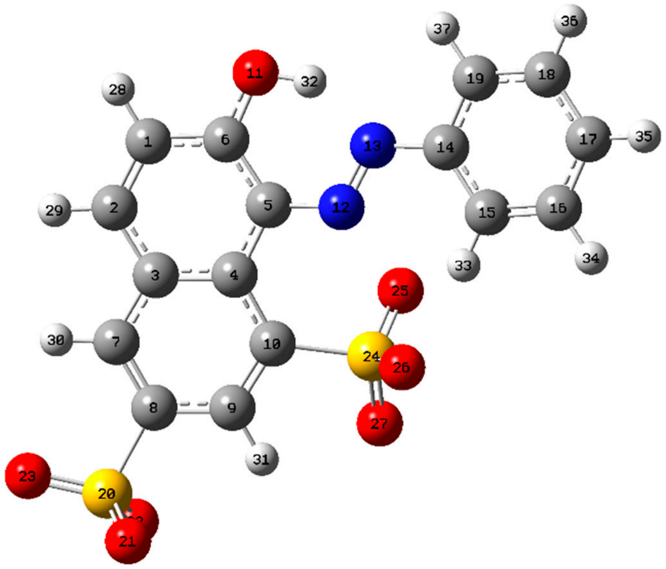

HETATM	1	C	0	0.108	3.713	0.222	C
HETATM	2	C	0	1.366	3.231	0.074	C
HETATM	3	C	0	1.623	1.835	0.017	C
HETATM	4	C	0	0.553	0.903	0.114	C
HETATM	5	C	0	-0.783	1.433	0.143	C
HETATM	6	C	0	-0.981	2.815	0.256	C
HETATM	7	C	0	2.958	1.408	-0.141	C
HETATM	8	C	0	3.249	0.079	-0.170	C
HETATM	9	C	0	2.227	-0.859	0.041	C
HETATM	10	C	0	0.917	-0.481	0.204	C
HETATM	11	O	0	-2.197	3.359	0.339	O
HETATM	12	N	0	-1.839	0.553	-0.100	N
HETATM	13	N	0	-2.992	0.997	0.084	N
HETATM	14	C	0	-4.069	0.124	-0.206	C
HETATM	15	C	0	-3.907	-1.233	-0.479	C
HETATM	16	C	0	-5.029	-2.005	-0.734	C
HETATM	17	C	0	-6.299	-1.435	-0.720	C
HETATM	18	C	0	-6.452	-0.083	-0.440	C
HETATM	19	C	0	-5.336	0.698	-0.177	C
HETATM	20	S	0	4.908	-0.511	-0.397	S
HETATM	21	O	0	4.862	-1.347	-1.593	O
HETATM	22	O	0	5.221	-1.277	0.804	O
HETATM	23	O	0	5.741	0.673	-0.558	O
HETATM	24	S	0	-0.172	-1.849	0.653	S
HETATM	25	O	0	-1.091	-1.346	1.664	O
HETATM	26	O	0	-0.813	-2.300	-0.576	O
HETATM	27	O	0	0.722	-2.866	1.196	O
HETATM	28	H	0	-0.101	4.771	0.284	H
HETATM	29	H	0	2.208	3.906	0.006	H
HETATM	30	H	0	3.736	2.153	-0.237	H
HETATM	31	H	0	2.485	-1.905	0.119	H
HETATM	32	H	0	-2.850	2.601	0.325	H
HETATM	33	H	0	-2.916	-1.662	-0.479	H
HETATM	34	H	0	-4.914	-3.059	-0.943	H
HETATM	35	H	0	-7.167	-2.047	-0.921	H
HETATM	36	H	0	-7.437	0.362	-0.424	H
HETATM	37	H	0	-5.431	1.752	0.049	H

END

Table S4. *FT-IR spectrum. Band assignment. Sample: carbonaceous adsorbent material (ACM)*

Wavenumber (cm^{-1})	Vibration	Group
3500	υ(O-H)	-OH
2932, 2847	υ(C-H)	$-CH_3$, $-CH_2-$
1729	υ(C=O)	Carboxylic acid
1607,1486	υ(C=C)	Aromatic ring
1499, 1447	υ(C=C)	Aromatic
1447, 1389	δ(C-H)	$-CH_3$, $-CH_2-$
	δ(O-H), υ(C=O)	Carbonyl
1350-1150	δ(S=O)	Sulfones
1350-1175	δ(S=O)	Sulphonates
1280	υ(=C-O)	OH phenolics
1123,1068	υ(C-O)	Ether-like structures
1050	δ(S=O)	Sulfoxide
900-700	γ(C-H)	Aromatic
700-400	υ(C-C)	Aliphatic

Abbreviations: υ, tension vibration; δ, deformation vibration (in-plane); γ, deformation vibration (out-of-plane).
